# Effective Non-invasive Treatment of Lingual Hematoma in an End-Stage Renal Disease (ESRD) Patient on Rivaroxaban: A Case Report

**DOI:** 10.7759/cureus.61581

**Published:** 2024-06-03

**Authors:** Abdelwahab Jalal Eldin, Nassar Mukhtar, Merawi Adane, Calbee Newkirk

**Affiliations:** 1 Internal Medicine, Wilson Medical Center, Wilson, USA; 2 Internal Medicine, Henrico Doctors’ Hospital, Richmond, USA; 3 Medicine, Wilson Medical Center, Wilson, USA

**Keywords:** end-stage renal disease (esrd), warfarin, atrial fibrillation, rivaroxaban, lingual hematoma

## Abstract

Lingual hematoma is a rare, life-threatening condition that can obstruct the airway. We report a 73-year-old male with end-stage renal disease (ESRD) who developed lingual hematoma while on rivaroxaban. He presented with odynophagia and significant tongue swelling. Treatment with vitamin K, dexamethasone, tranexamic acid, and prothrombin complex concentrate led to rapid improvement without the need for intubation. This case highlights the importance of prompt medical management to prevent airway obstruction in similar patients.

## Introduction

Lingual hematoma, though rare, is a potentially fatal condition if not recognized early, as it can quickly compromise the airway [1]. While there have been only a few documented cases of lingual hematoma, none have been reported in patients on rivaroxaban with end-stage renal disease [2]. Currently, there are no guidelines on the medical treatment of a developing lingual hematoma [3,4]. In this report, we highlight a compelling case of lingual hematoma in an end-stage renal disease (ESRD) patient on rivaroxaban for atrial fibrillation (AFib), successfully managed medically, thereby averting the need for intubation.

## Case presentation

A 73-year-old male presented with painful swallowing, significant tongue swelling, and redness for one day, alongside a left arm bruise. He recently switched from apixaban to rivaroxaban (20 mg) for AFib due to insurance changes and had taken rivaroxaban before arrival. The day before, he had visited the emergency room with a sore throat and was discharged with cephalexin. There were no prior instances of tongue swelling, and he wasn't using angiotensin-converting enzyme inhibitors or blockers. Although a fall occurred a week prior, resulting in an arm bruise, both the patient and his caregiver denied any history of head or tongue trauma. They only noticed the oral symptoms six days after the fall, a day before presentation.

**Figure 1:**
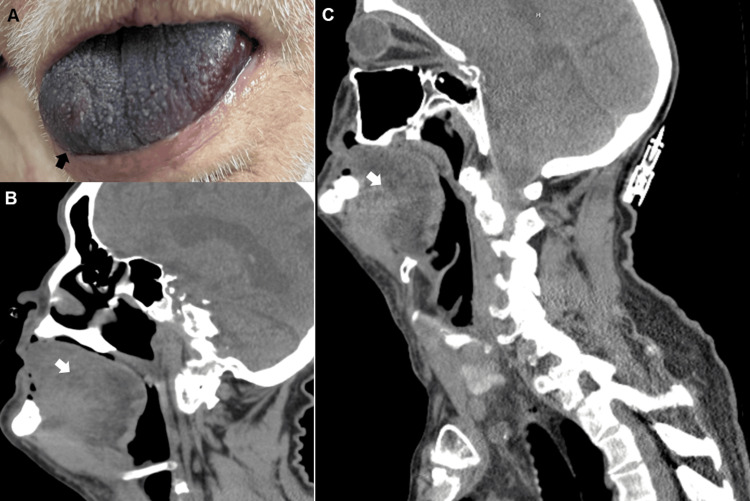
(A) Lingual hematoma. (B and C) Diffuse swelling of the tongue extending to the base of the tongue

His medical history includes ESRD treated with peritoneal dialysis, insulin-dependent diabetes, coronary artery disease with prior coronary artery bypass graft, dyslipidemia, primary hypertension, hypothyroidism, and non-Hodgkin's lymphoma in remission. Vital signs indicated a blood pressure of 192/86 mmHg, heart rate of 70 bpm, respiratory rate of 14 breaths per minute, and temperature of 98.6°F. Physical examination revealed a significantly swollen, cyanotic tongue (Figure 1A). Investigations showed an elevated international normalized ratio (INR) of 3.2, an elevated APTT of 68.8, and anemia (Table 1). Contrast CT neck showed diffuse swelling of the tongue extending to the base of the tongue with no evidence of an abscess, alongside emphysematous changes at the lung apices with nodular opacities in the right upper lobe indicative of potential superimposed infection (Figures 1B, 1C). Both COVID and influenza PCR tests were negative.

**Table 1:** Laboratory investigations WBC: white blood count; INR: international normalized ratio; APTT: activated partial thromboplastin time; ALT: alanine transaminase.

Investigation	Result on admission	Reference
WBC	22.4	3.8-10.7 × 10^9^/L
Hemoglobin	11.5	12-17.5 g/dL
Hematocrit	34.9	35.8-52.9 %
Platelet	458	150-450 × 10^9^/L
INR	3.2	
APTT	68.8	23.9-37.6 seconds
ALT	12	0-50 U/L
Lactic acid	2	0.5-2 mmol/L

Treatment in the ER included intramuscular vitamin K (5 mg), intravenous dexamethasone (8 mg), intravenous tranexamic acid (one gram), and ceftriaxone (one gram). He was admitted to the medical ICU for monitoring and received two grams of prothrombin complex concentrate. Swallowing improved within hours, and tongue swelling gradually resolved during hospitalization. No intubation was required, and he was discharged after three days.

## Discussion

Lingual hematoma, although rare, poses a significant threat to life due to its potential to obstruct the airway [1]. The tongue, abundantly supplied by the lingual artery, possesses a rich blood supply, rendering it susceptible to bleeding [5]. In this case, the use of rivaroxaban in the context of ESRD was a primary factor in the hematoma’s development, with a possible but less likely contribution from a mechanical fall one week prior. Given the absence of established guidelines for managing lingual hematoma, reliance on case reports currently serves as a cornerstone for guiding clinical decisions [3,4].

Current guidelines advise against prescribing rivaroxaban for stroke prevention in AFib patients with ESRD undergoing dialysis. This caution stems from the scarcity of robust evidence demonstrating that the benefits of rivaroxaban outweigh the risks in this population. Studies have indicated that rivaroxaban use in hemodialysis patients elevates the risk of bleeding-related adverse events compared to warfarin [6,7].

The danger of lingual hematoma lies in its capacity to swiftly obstruct the airway, which could be fatal without prompt intervention. While our patient sought medical attention promptly within 24 hours of symptom onset, the potential ramifications of delayed intervention remain uncertain. In cases of rapid hematoma progression, securing the airway becomes imperative for patient survival. Endotracheal intubation is likely not feasible due to oral obstruction and the risk of airway bleeding during manipulation [8]. Nasotracheal intubation, though an option, entails higher risks and demands specialized expertise, typically involving an anesthesiologist. Alternatively, cricothyroidotomy offers a quicker means to bypass the upper airway obstruction, albeit with an increased potential for complications [4,9].

Aggressive medical management of lingual hematoma offers a non-invasive approach to mitigate swelling and halt hematoma progression, potentially averting invasive measures such as intubation [10]. Our patient, with a supratherapeutic INR of 3.2, received vitamin K, dexamethasone, tranexamic acid, and prothrombin complex concentrate. Although the optimal medical approach remains unclear, our case underscores the efficacy of proactive medical management in mitigating the impending threat of airway compromise.

In cases where airway obstruction becomes imminent, securing the airway becomes unavoidable. Thus, transitioning the patient to a higher level of care can be lifesaving if advanced airway intervention becomes necessary during medical treatment [1]. Despite our patient's hematoma rapidly progressing within a day, the absence of distress necessitating urgent intubation, along with clinical improvement with the medications used, highlights the efficacy of the chosen medical management strategy.

Further studies are needed to delineate which medical treatment offers superior efficacy in managing lingual hematoma.

## Conclusions

This case report highlights the successful medical management of a lingual hematoma in a 73-year-old ESRD patient on rivaroxaban. Prompt treatment with vitamin K, dexamethasone, tranexamic acid, and prothrombin complex concentrate prevented airway compromise and the need for intubation. It underscores the importance of early intervention and the need for further research to establish guidelines for managing this rare condition.
